# Global Hotspots of Whale–Ship Collision Risk: A Multi-Species Framework Integrating Critical Habitat Zonation and Shipping Pressure for Conservation Prioritization

**DOI:** 10.3390/ani15142144

**Published:** 2025-07-20

**Authors:** Bei Wang, Linlin Zhao, Tong Lu, Linjie Li, Tingting Li, Bailin Cong, Shenghao Liu

**Affiliations:** 1 Marine Ecology Research Center, First Institute of Oceanography, Ministry of Natural Resources, Qingdao 266061, China; wangbei@fio.org.cn (B.W.); zhaolinlin@fio.org.cn (L.Z.); lutong@fio.org.cn (T.L.); ytlitingting@163.com (T.L.); biolin@fio.org.cn (B.C.); 2 Observation and Research Station of Bohai Strait Eco-Corridor, First Institute of Oceanography, Ministry of Natural Resources, Qingdao 266061, China; 3 Department of Fisheries, Ocean University of China, Qingdao 266003, China; aa571863785@163.com

**Keywords:** collision hotspot, shipping environmental impact, species distribution modeling, endangered whale species, marine protected areas, spatial planning

## Abstract

The expansion of global maritime activities threatens marine ecosystems and biodiversity. Collisions between ships and marine megafauna profoundly impact vulnerable species such as whales, who serve as keystone predators. However, the specific regions most heavily affected by shipping traffic and the multi-species facing collision risk remain poorly understood. Here, we analyzed global shipping data to assess the distribution of areas with high shipping pressure and identify global hotspots for whale–ship collisions. The results reveal that high-pressure habitats are primarily distributed within exclusive economic zones (EEZs), which are generally consistent with the distribution of collision hotspots. High-pressure habitats exhibit significant spatial mismatch: 32.9% of Marine Protected Areas endure high shipping stress and yet occupy merely 1.25% of protected ocean area. Additionally, 25.1% of collision hotspots (top 1% risk) affect four or more whale species, forming critical aggregation in regions like the Gulf of St. Lawrence and Northeast Asian marginal seas. Most of these high-risk areas lack protective measures. These findings offer actionable spatial priorities for implementing targeted conservation strategies, such as the introduction of mandatory speed restrictions and dynamic vessel routing in high-risk, multi-species hotspots. By focusing on critical aggregation areas, these strategies will help mitigate whale mortality and enhance marine biodiversity protection, supporting the sustainable coexistence of maritime activities with vulnerable marine megafauna.

## Simple Summary

The expansion of global maritime activities threatens marine ecosystems, particularly through ship collisions with vulnerable keystone species like whales, though the most impacted regions and species remain unclear. This study analyzed global shipping data to identify areas of high shipping pressure and whale–ship collision hotspots. The results show high shipping pressure and collision risk primarily occur within coastal waters. Furthermore, critical collision hotspots posing risks to multiple whale species simultaneously were identified in regions such as the Gulf of St. Lawrence and Northeast Asian seas. However, most of these high-risk areas currently lack protective measures. These findings provide crucial spatial priorities for targeted conservation strategies, such as mandatory speed restrictions and dynamic vessel routing in critical multi-species hotspots. Focusing interventions on these key areas can help mitigate whale mortality, enhance marine biodiversity protection, and support the sustainable coexistence of shipping and vulnerable marine megafauna.

## 1. Introduction

The rapid expansion of maritime trade networks poses escalating threats to marine ecosystems in an increasingly globalized economy. In 2020, the global commercial shipping fleet grew by 3%, reaching 99,800 ships of 100 gross tons and above. By January 2021, their capacity was equivalent to 2.13 billion dead weight tons (dwt), and approximately 90% of globally traded goods were transported via maritime routes [1]. Shipping volumes have surged and quadrupled since 1992, and they are projected to grow further in the coming decades [2]. This growth intensifies risks to marine megafauna [3,4,5,6,7], including accelerating climate change [8], causing chemical and noise pollution [3,9], inducing species transfer or species invasion [10], and resulting in a disruption to critical biological processes such as the survival, reproduction, and migration of marine organisms [11], which could potentially cause a loss of biodiversity and raises concerns about potential impacts on marine fauna. The rising incidence of fatal marine wildlife collisions with vessels has emerged as a critical conservation challenge. These interactions frequently result in severe injury or mortality for the marine organisms, while concurrently endangering vessel integrity and human safety. Current analyses integrating necropsy records and collision reports reveal that over 75 marine species face collision risks [7], encompassing endangered cetaceans (North Atlantic right whales, fin whales, blue whales, humpback whales, sperm whales), marine reptiles (sea turtles), and sirenians (Florida manatees). However, there is a lack of comprehensive identifications of the specific geographic hotspots where whale–ship collisions are most frequent, which hinders the development of targeted conservation strategies and shipping route modifications.

Marine Protected Areas (MPAs) are mainly located in marine areas under national jurisdiction, and they are established to protect biodiversity and marine ecosystems [12]. Ecologically or biologically significant marine areas (EBSAs) were proposed by the Convention on Biological Diversity (CBD) to prioritize areas in the ocean for conservation using seven scientific criteria [13]. Additionally, Important Marine Mammal Areas (IMMAs) are initiatives for the establishment of important mammal conservation areas, mainly based on the vulnerability of species or populations, their distribution and abundance, their vital life cycle, and their uniqueness and diversity as the criteria for selection and delineation [14]. These areas are typically chosen for their important and endangered species and encompass diverse habitat types, ensuring the inclusion of most critical species habitats. Currently, only 8.36% of marine areas are protected, with only 2.8% fully protected [15]. EBSAs and IMMAs also lack mandatory management measures and are vulnerable to the negative impacts of shipping activities. The recent adoption of the UN Agreement on Marine Biodiversity Beyond National Jurisdiction reflects a global consensus for balancing sustainable resource use with biodiversity conservation. However, there is an insufficient integration of shipping pressure data with critical habitat zonation, which limits the ability to prioritize conservation efforts in areas where shipping activities pose the greatest threat to biodiversity.

Large whales play crucial roles in marine ecosystems, including the top-down and bottom-up regulation of marine food webs, nutrient cycling, and transport [16]. As flagship species in the ocean, whales face severe direct vessel strikes as their primary anthropogenic threat [7,17]. Recent spatial assessments demonstrate that 92% of whale habitats overlap with active shipping lanes, yet merely 7% of collision hotspots—defined as the highest-risk zones (top 1% collision probability)—implement protective measures [18]. For critically endangered megafauna with small, slow-moving populations inhabiting surface waters in high-traffic regions, lethal collisions constitute a primary impediment to population recovery [19]. Post-industrial whaling era whale populations, already reduced to fractions of their historical abundance, have become even more vulnerable [20]. Currently, ship strikes pose a serious threat to whales globally, resulting in higher anthropogenic mortality rates than legally permitted for some populations [21], contributing to the decline of critically endangered species. Assessing the risk of collisions between animals and ships is an important step in implementing mitigation measures in relevant geographic areas [22]. However, there is currently a lack of collision risk assessment measures that integrate multi-species distribution information. Prioritizing sites for ship collision management through risk assessment can help protect endangered species, reduce the risk of species loss, and minimize ecological damage.

This study assessed the pressures of shipping on biologically important marine areas and selected nine whale species exhibiting heightened collision susceptibility for targeted risk assessment. By assessing the effectiveness of current collision management zones, we found that high-risk regions remain inadequately managed, with substantial spatial overlap between shipping routes and critical whale habitats. Our findings offer a framework for marine spatial planning, recommending speed limits and dynamic route adjustments in multi-species hotspots. These approaches can mitigate whale mortality while fostering sustainable maritime transport, aiding policymakers in balancing economic development with ecological conservation.

## 2. Materials and Methods

### 2.1. Data Collection

**Figure 1:**
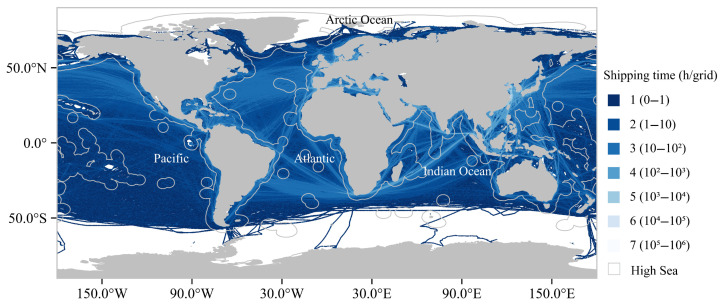
Schematic of global shipping hours in 2023.

We processed global maritime traffic patterns using hourly vessel tracking data (December 2024) for large vessels equipped with AIS system from Global Fishing Watch (https://globalfishingwatch.org/, accessed on 29 December 2024). These data were aggregated into a 0.1° × 0.1° grid system and classified into eight intensity levels (Figure 1) via logarithmic scaling (1–3: low intensity; 4: moderate intensity; 5–7: high intensity; criteria detailed in Table S1 and Text S1). MPA boundaries were sourced from Protected Planet (https://www.protectedplanet.net/en, accessed on 12 September 2024), retaining only exclusively marine MPAs and those with mixed marine–terrestrial compositions where marine areas exceeded 50% of total coverage (*n* = 10,378; Figure S1A). EBSAs were obtained from the Convention on Biological Diversity (https://www.cbd.int/ebsa, accessed on 7 June 2024; *n* = 336; Figure S1B), while IMMAs were acquired from the Marine Mammal Protected Areas Task Force (https://www.marinemammalhabitat.org, accessed on 26 February 2024; *n* = 280; Figure S1C). High-seas boundary data were from the Flanders Marine Institute [23]. High-seas jurisdictional boundaries were derived from the Flanders Marine Institute’s maritime zone database. Spatial analyses integrated these datasets to quantify shipping pressures across critical marine habitats.

**Figure 2:**
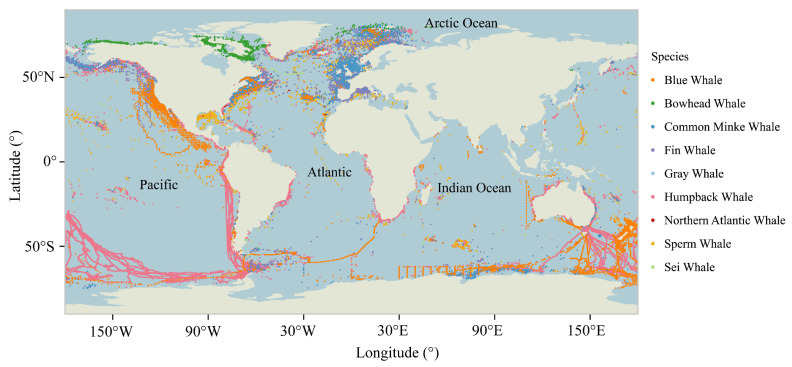
Global distribution of nine whale species.

Whale distribution records were obtained primarily from three public databases: the Ocean Biogeographic Information System (OBIS, https://obis.org), the Global Biodiversity Information Facility (GBIF, https://www.gbif.org) (GBIF, 2024), and OBIS-SEAMAP (https://seamap.env.duke.edu/). To enhance dataset completeness, we conducted a systematic literature review of peer-reviewed studies (1993.1–2024.10) indexed in the Web of Science database. Initial searches employed species-specific scientific nomenclature, followed by supplementary queries using common names to identify publications containing spatially explicit occurrence records. When geographic coordinates were not directly provided in the identified studies, we contacted their corresponding authors to request supplementary data. All collected records were temporally restricted to post-1993 observations to maintain temporal alignment with the subsequently analyzed environmental datasets. To minimize sampling bias in the occurrence data, we spatially thinned the occurrence data using the R (version 4.3.2) package spThin, and only one occurrence was retained in each grid cell, consistent with the spatial resolution of the environmental predictors [24]. After further filtering with species ranges assessed in the IUCN, a total of 23,108 validated occurrence points remained for the nine species (Figure 2).

Based on previous relevant studies [25,26,27], we initially selected 11 environmental variables that may affect whale distribution: sea surface temperature (SST), salinity (Sal), chlorophyll (Chl), mixed layer depth (MLD), sea ice thickness (SIT), sea surface height (SSH), sea ice cover (SIC), bathymetry (Bat), terrain slope (TS), terrain aspect (TA), and distance from shore (DS) (Table 1). All variables were resampled to a uniform resolution of 0.25° for subsequent analysis. To determine potential covariance between the predictor variables, Pearson correlation coefficients (r) were calculated between them [28]. Variables with high correlations (|r| > 0.7) were removed (Figure S1). Nine variables, including SST, Sal, Chl, MLD, SIT, Bat, TS, TA, and DS, were retained for subsequent modeling analyses (Table 1).

### 2.2. Model Construction

We implemented a stacked species distribution modeling framework using the R (version 4.3.2) package SSDM to predict habitat suitability for the nine whale species that proved to be vulnerable to ship collisions and were listed in the IUCN Red List [7,31]. This method enables concurrent multi-species distribution projections and assemblage pattern analysis, offering advantages over single-species modeling approaches like those in the “sdm” and “biomod2” packages [32,33]. The package includes nine algorithms: a generalized additive model (GAM), generalized linear model (GLM), multivariate adaptive regression spline (MARS), classification tree analysis (CTA), generalized boosting model (GBM), maximum entropy (MAXENT), artificial neural network (ANN), random forest (RF), and support vector machines (SVMs). To address data limitations, we applied Barbet-Massin et al.’s (2012) pseudo-absence generation method embedded in SSDM [34]. Model performance was evaluated using a fivefold cross-validation that was repeated ten times to improve robustness. This method randomly divided the data into a 4:1 ratio, allocating 80% for training and 20% for validation [35]. Predictive performance was evaluated using the Area Under the Curve (AUC) and True Skill Statistic (TSS) metrics, with reliability thresholds set at >0.8 and >0.7, respectively [36]. The AUC was calculated based on the test data retained during the cross-validation period, the TSS was calculated as the sensitivity (correctly predicted points of presence) plus the specificity (correctly predicted of missing or background points) minus 1. We created a weighted ensemble model incorporating only high-performance projections (AUC > 0.9) to reduce prediction uncertainty [37]. The final analysis quantified environmental variable contributions to species distributions using SSDM’s built-in ranking methodology [38].

### 2.3. Data Analysis

Geographic data for MPAs, EBSAs, and IMMAs were categorized into point and polygon types. For point data, the maximum value of the shipping hourly data in the eight grids closest to the point location was used. For polygon data, if the latitude and longitude range of the polygon was less than 0.1° × 0.1°, the polygon was simplified to be processed as point data. If the latitude/longitude range of the polygon was greater than 0.1° × 0.1°, the average of all shipping hour data within the boundary was used. Incomplete maritime traffic coverage resulted in excluded records, yielding 7688 valid MPAs (3537 points, 4151 polygons), 319 EBSAs, and 268 IMMAs for analysis. We assumed that there were no MPAs in the high seas because the vast majority of MPAs were located within EEZs. EBSA and IMMA jurisdictional assignments were determined through boundary analysis: features intersecting high-seas boundaries were classified as transboundary, while fully EEZ-contained features maintained national jurisdiction status.

The stacked species distribution model (SSDM) generated both binary and continuous probability projections for the nine whale species. The spatial aggregation of binary outputs enabled an analysis of global cetacean abundance patterns. For collision risk assessment, we processed global shipping intensity by applying logarithmic transformation (minimum threshold = 10) followed by 0–1 normalization to create a shipping density index *Vi.* The whale presence probability was derived from continuous distribution models and was noted as *Di*. The collision risk Ri was defined as the product of *Vi* and *Di*, i.e.,

Ri=Vi×Di (i stands for grid)

Ship collision hotspots were defined as grid cells exceeding the 99th percentage of collision risk values (top 1% risk) for each species. We also analyzed areas with risks greater than or equal to 90%, 95%, 99%, and 99.5% to assess differences in collision hotspot areas across species. We overlayed risk hotspots for each species to identify areas that pose a high collision risk for multiple species.

## 3. Results

### 3.1. High-Pressure Critical Habitats Primarily Found Offshore and Vulnerable to Shipping Stress

**Figure 3:**
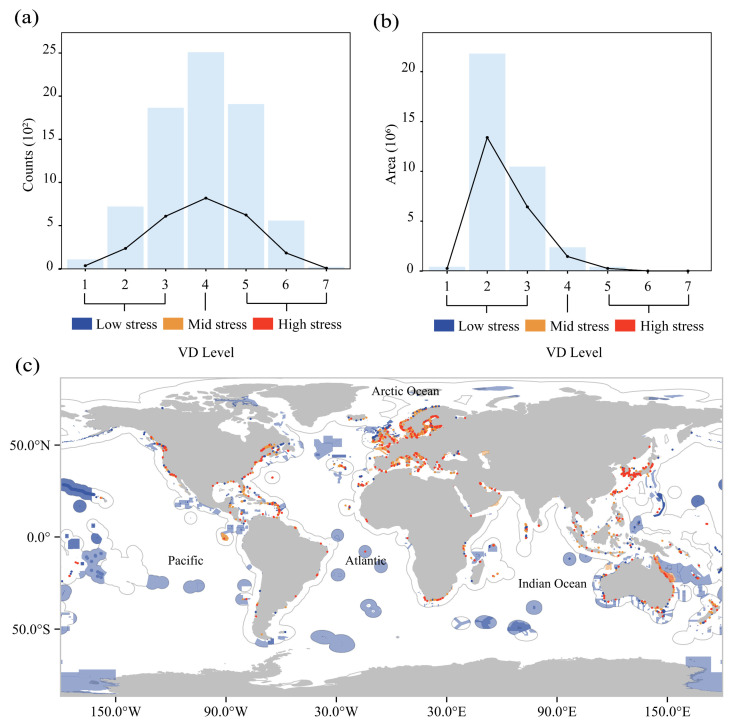
Number and area of MPAs under shipping stress and global distribution of MPAs. (**a**): By number; (**b**): by area; (**c**): showing global distribution of MPAs at different levels of shipping stress. Blue, yellow, and red represent low, medium, and high shipping pressure, respectively. Levels 1–3 represent low shipping pressure, level 4 represents medium shipping pressure, and levels 5–7 represent high shipping pressure.

MPAs exhibited distinct spatial patterns under seven-tiered shipping intensity gradients (Figure 3a,c). Numerically, the MPAs demonstrated a near-equal distribution across the low- (33.90%), moderate- (33.17%), and high-stress (32.94%) categories. However, spatial coverage revealed significant disparities: low-stress MPAs (levels 1–3) encompassed 92.06% of the total protected ocean area, moderate-stress MPAs (level 4) accounted for 6.69%, and high-stress MPAs (levels 5–7) represented merely 1.25% (Figure 3b). Geospatial analysis identified concentrated high-intensity zones in coastal regions, particularly along the Japanese and Korean peninsulas, as well as European maritime territories.

**Figure 4:**
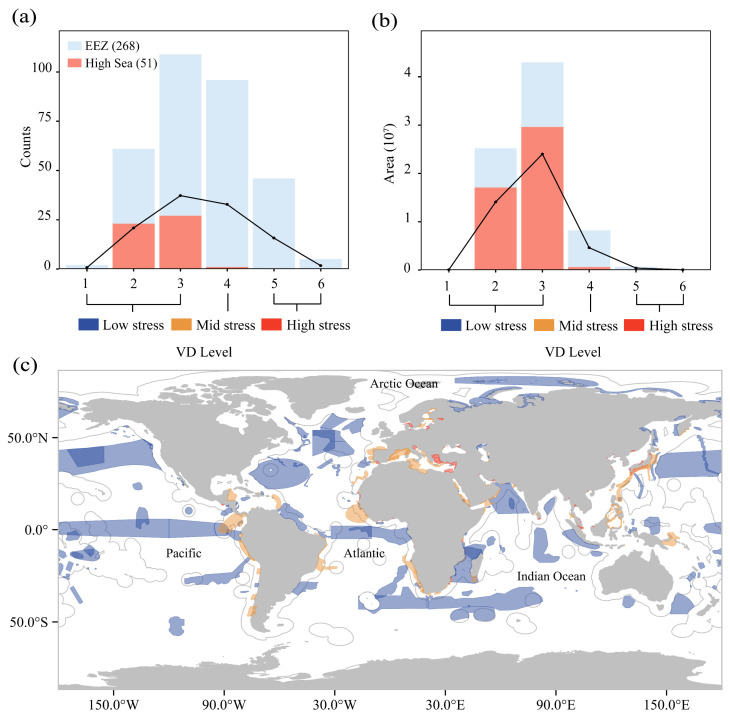
Number and area of EBSAs under shipping stress and global distribution of EBSAs. (**a**): By number; (**b**): by area; (**c**): showing global distribution of EBSAs at different levels of shipping stress. Blue, yellow, and red represent low, medium, and high shipping pressure, respectively. Levels 1–3 represent low shipping pressure, level 4 represents medium shipping pressure, and levels 5–7 represent high shipping pressure.

EBSAs exhibited maximum shipping stress at level 6, with 2, 61, 109, 96, 46, and 5 EBSAs categorized across stress levels 1–6 (0.63%, 19.12%, 34.17%, 30.09%, 14.42%, and 1.57% of total EBSAs, respectively; (Figure 4a,b)). High-seas EBSAs were concentrated in stress levels 2–3 (45.10% and 52.94%), while no high-seas EBSAs occurred in higher stress categories (levels 4–6). Overall, 84% of EBSAs experienced low-to-moderate shipping pressure, with only 51 high-stress EBSAs (levels 5, 6) located exclusively within EEZs. The high-stress areas were mainly located in the Mediterranean Sea, the North Sea, and the Baltic Sea (Figure 4c).

**Figure 5:**
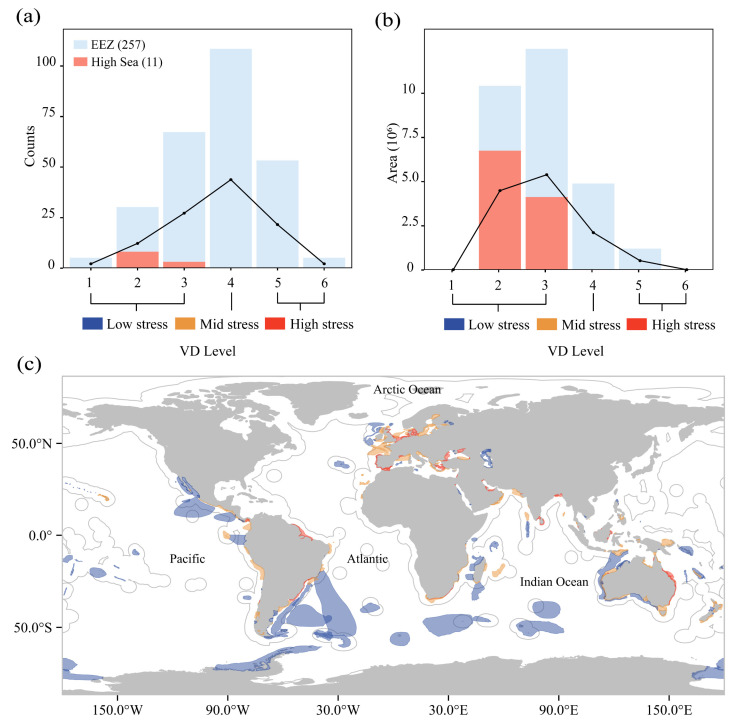
Number and area of IMMAs under shipping stress and global distribution of IMMAs. (**a**): By number; (**b**): by area; (**c**): showing global distribution of IMMAs at different levels of shipping stress. Blue, yellow, and red represent low, medium, and high shipping pressure, respectively. Levels 1–3 represent low shipping pressure, level 4 represents medium shipping pressure, and levels 5–7 represent high shipping pressure.

There were 5 IMMAs in classes of which only 4.10% (8 level 2, 3 level 3) were located within high seas (Figure 5a). The proportion of IMMAs under low, medium, and high shipping stress was 38.06%, 40.30%, and 21.65%, respectively. Most of the IMMAs were under medium shipping stress. High-stress IMMAs were predominantly concentrated in coastal EEZs, notably within the Mediterranean Sea and Malaysian and Chinese coastal waters (Figure 5c).

### 3.2. Ship Strike Risks to Cetaceans Reveal Global Hotspots and Species-Specific Vulnerabilities

The ensemble models demonstrated robust predictive accuracy across all species, with evaluation metrics (AUC > 0.9, TSS > 0.7) confirming high model reliability in habitat suitability projections (Table S2). Variable importance analysis revealed distance from shore and sea surface temperature (SST) as primary determinants of habitat suitability for six whale species. Bathymetry and chlorophyll concentration emerged as key drivers for North Atlantic right whale distributions, while gray whale habitat selection showed its strongest associations with chlorophyll concentration and salinity. Bowhead whales demonstrated unique ecological dependencies, with sea ice cover constituting the predominant distributional constraint (Figure S3).

**Figure 6:**
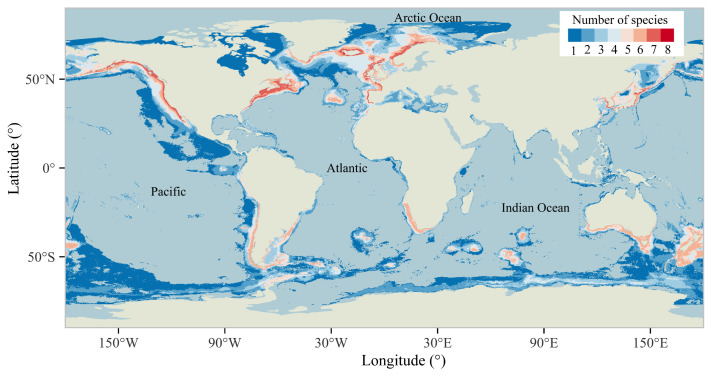
Species richness distribution for whales. From dark blue to bright red represents an increase in the number of species.

Habitat suitability projections (Figure S4) revealed both shared and different distributional ranges across the nine whale species. While blue, minke, humpback, and sperm whales demonstrated a near-global distribution, distinct regional specializations emerged: blue whales dominated North America’s western coastal waters and Antarctic regions, minke and sei whales concentrated in the North Atlantic, and humpback whales occupied the expansive South Pacific. The binary distributions of all species are superimposed to show the distribution of species richness (Figure 6), with coastal zones exhibiting significantly higher cetacean diversity than oceanic interiors. Areas of high species richness were concentrated in the North Atlantic, Gulf of Alaska, Gulf of Maine, Gulf of St. Lawrence, and east coast of Japan, where more than six of the nine whale species were distributed (Figure 6).

We analyzed areas with risks greater than or equal to 90%, 95%, 99%, and 99.5% to assess differences in collision hotspot areas across species. The results revealed globally widespread ship strike threats to cetaceans, with distinct geospatial concentrations across species (Figure S5). All ocean areas presented a significant ship collision risk to whales. Blue, minke, fin, humpback, sei, and sperm whales shared collision hotspots notably prevalent along the North American Pacific coast, Gulf of St. Lawrence, European shelf seas (Norway, Iberian Peninsula), southern African waters, Sri Lankan coastal zone, Australian southern shelf, and Northeast Asian marginal seas (Yellow/Bohai Seas, Japanese coastal waters). Specialized risk patterns emerged for particular taxa: Mediterranean waters showed high strike probabilities for minke, fin, and sperm whales; bowhead whale hotspots clustered in Baffin Bay; gray whale high-risk zones centered on Japanese and Chinese coastal EEZs; and North Atlantic right whales faced critical threats in the Gulf of St. Lawrence, U.S. eastern seaboard, and Norwegian/Portuguese shelf waters.

**Figure 7:**
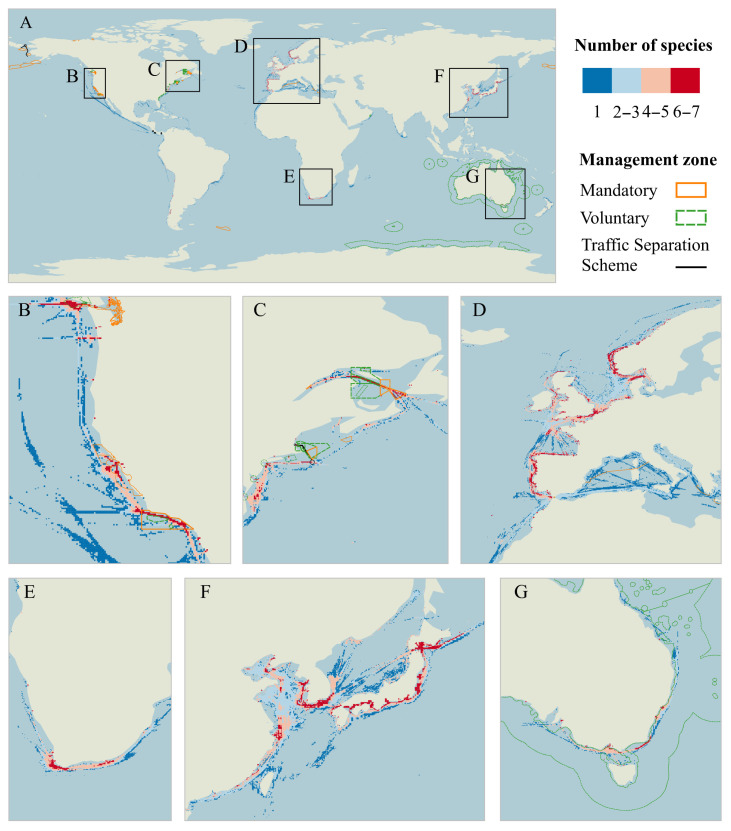
Ship collision hotspots for whales. (**A**): Spatial overlap of collision hotspots for nine species of whales. Hotspots are defined as areas in the top 1% of ship strike risk for each species. (**B**–**G**) show zoomed-in maps of localized areas. (**B**) West coast of North America, (**C**) east coast of North America, (**D**) eastern North Atlantic Ocean, (**E**) southern waters of South America, (**F**) western North Pacific Ocean, and (**G**) east coast of Australia.

Collision hotspots zones (top 1% risk percentile) were identified through the spatial aggregation of species risk maps, revealing concentrated threats along coastal waters with notable exceptions in pelagic regions (Figure 7). This is consistent with the results of the analysis of the pressure exerted by shipping on the habitats of important species. While the coastal waters of the western North and eastern North Pacific, Gulf of St. Lawrence, North Atlantic, and southern Africa emerged as primary multi-species risk clusters (>6 species affected), secondary hotspots occurred in the Mediterranean Sea, eastern North Atlantic, and southeastern Australian waters.

## 4. Discussion

As the main currency for international trade [39], maritime shipping drives economic growth but also threatens marine ecosystems through multiple pressures [3,8,10]. Identifying areas of intense shipping activity and whale collision risks using species distribution data is crucial for protecting critical habitats and creating effective management plans. Our research demonstrates how shipping routes overlap with sensitive marine habitats, showing human impacts on key ecological zones. By mapping existing whale management areas, our study provides practical strategies to improve conservation efforts where shipping activity and whale habitats intersect most significantly.

### 4.1. High-Pressure Critical Habitats Found Primarily Offshore

Global maritime traffic exhibits pronounced spatial clustering, with the Northwest Pacific, Indian Ocean, and Northeast Atlantic having high-density shipping routes, while polar regions (Southern and Arctic Oceans) remain minimally traversed. This uneven distribution concentrates anthropogenic pressures in the coastal zones of the Northwest Pacific and Northeast Atlantic, where MPAs are predominantly located within EEZs. MPAs are used to protect the flora, fauna, and historical and cultural attributes of the area [40]. Coastal shipping lanes serve as critical connectors between port infrastructure and global trade networks [41], with concentrated maritime activity in economic hubs like Europe’s North Sea and China’s Pearl River Delta creating overlapping zones of ecological sensitivity and intensive vessel traffic. These regions within MPAs experience graded shipping pressures, while Areas Beyond National Jurisdiction (ABNJs) currently lack high-pressure MPAs due to limited maritime operations. The difference between the numerical equality and areal dominance of low-stress MPAs emphasizes the severe vulnerability of limited but ecologically significant nearshore protected areas to intensive shipping activities. However, expanding port development and transoceanic route optimization under trade globalization threaten to accelerate environmental degradation in high-seas ecosystems through multiple pathways: chemical and noise pollution [42], greenhouse gas emissions [43], species invasion risks [44], and seabed habitat destruction from deep-sea mining [45]. This impending pressure surge necessitates expanded conservation assessments beyond EEZ-focused MPAs to incorporate EBSAs and IMMAs in international waters, ensuring the proactive management of anthropogenic impacts across jurisdictional boundaries.

The concentration of six high-pressure EBSAs within EEZs highlights the urgent need for the enhanced jurisdictional management of these coastal habitats. While high-seas EBSAs currently exhibit moderate-to-low shipping pressures, their vast spatial extents may obscure localized high-intensity impacts when assessed through spatial averaging methods. EBSAs are usually used to protect specific species, sensitive ecosystems, or areas of high naturalness. These offshore areas have critical ecological functions, including migratory corridors for cetaceans and seabirds, vulnerable ecosystems (seagrass beds, mangroves, coral reefs), and endemic species such as dugongs. Notably, the NWIO_3 EBSA hosts Dubai’s sole remaining nesting site for critically endangered hawksbill turtles, exemplifying their irreplaceable conservation value. Sea turtles, recognized as vital indicator species [46], face direct threats from maritime operations in critical habitats like EA_12 and NWIO_03, where propeller strikes and pollution compromise their survival [7,47]. Shipping-derived pressures extend beyond physical harm: ballast water mediates invasive species transfer, exemplified by the Spartina alterniflora invasion in EA_06 that disrupted subtropical wetland ecosystems [48]. Noise pollution from vessel traffic in BSCS_10, a key cetacean habitat, interferes with marine mammal communication and navigation, elevating ship strike risks [11]. Essential coastal ecosystems (seagrass beds, coral reefs, and mangroves) sustain biodiversity hotspots for dugongs, seabirds, and dolphins, yet accumulate pollutants that are biomagnified through food webs, threatening species health [49,50,51]. These multifaceted impacts, spanning physical, chemical, and biological stressors, resist simplistic quantification, necessitating enhanced monitoring and adaptive management frameworks to mitigate cumulative ecological damage in vulnerable marine zones.

Maritime activities pose dual threats to cetaceans through direct physical collisions and indirect ecological disruptions, exacerbated by the spatial overlap between shipping lanes and surfacing zones critical for marine mammal respiration [17]. Chronic vessel noise induces habitat displacement in dolphin populations [52], interferes with sonar systems, causes cetacean strandings [53], and imposes chronic stress, reducing reproductive success [54]. These impacts are particularly acute in five high-pressure IMMAs sheltering vulnerable species like the *Delphinus delphis* and *Sotalia guianensis*. As top predators, cetacean population declines trigger trophic cascades: mid-water fish proliferation pressures benthic ecosystems, while unchecked cephalopod populations degrade coral reef structures. Continuous ship noise, which causes dolphins to move away, may lead to a decline in the ecological function of their original habitat, and thus a decline in biodiversity in localized marine areas.

Given increasing maritime expansion, the advanced management of high-risk zones, implementing acoustic buffer areas and dynamic traffic regulation, becomes imperative to mediate ecological integrity with maritime economic priorities through evidence-based spatial planning.

### 4.2. Most Collision Risk Hotspots Have Not Implemented Risk Mitigation Measures

Using whales as indicator species, this study validated the proposed pressure assessment framework by analyzing habitats under intense maritime pressures. Habitat suitability modeling revealed concentrated whale distributions in coastal zones and the North Atlantic (Figure 5), with significant spatial overlap between high-probability habitats and intensive shipping corridors, elevating collision risks. Some open seas, particularly near the Azores archipelago, demonstrated high strike probabilities for blue, minke, fin, sei, and sperm whales. Polar regions exhibited minimal collision risks, with Arctic zones containing limited hotspots and the Southern Ocean having no hotspots due to negligible maritime traffic. Hotspot heterogeneity reflects species-specific ecological preferences and regional traffic densities, particularly in semi-enclosed basins and continental shelves where shipping lanes intersect critical cetacean habitats. Multi-species analysis identified 15.7% of hotspots as impacting three species, 9.0% affecting four species, and 14.1% influencing over four species (Table S3), demonstrating the efficacy of multi-species mitigation strategies. High-overlap zones require prioritized speed reduction measures, while route adjustments require balancing multi-species habitat distributions. It has been shown that the risk of collision can be reduced by 60–95% when compliance with a routing measure is high [55,56,57]. These findings establish a framework for balancing maritime operations with megafauna conservation in ecologically sensitive seascapes.

The International Whaling Commission’s (IWC) regional-scale collision risk assessments align with our global analysis [58], confirming the high-risk zones for blue whales (Sri Lanka, eastern North Pacific), sperm whales (Mediterranean), and sei whales (Mediterranean) (Figure S5). Our study further identifies underrecognized high-risk areas in the open oceanic regions (Azores, Mauritius, Réunion) and coastal waters of South African countries such as Madagascar, Mozambique, and Comoros, providing critical baseline data for targeted conservation. However, further field surveys and observations are needed for more contextualized management measures. Climate-driven Arctic Sea ice retreat and projected cetacean poleward migrations may lead to an increased collision probability, which necessitates the proactive monitoring of emerging Arctic shipping corridors [27,59]. Concurrently, the Southern Ocean’s low-shipping zones, harboring abundant blue and minke whale populations, represent vital climate refuges requiring enhanced protection frameworks.

Existing whale protection frameworks predominantly feature mandatory and voluntary vessel regulations along North American coastlines, employing speed restrictions (<10 knots), fishing prohibitions, and 400 m whale approach distances [60]. While most regulated zones exhibit low collision risks, persistent vulnerabilities occur in voluntary compliance areas along the Pacific Northwest (San Francisco–Monterey Bay corridor, Southern California coastal waters, Southern British Columbia), suggesting inadequate enforcement efficacy. This should be considered to enhance management or change it to mandatory areas. High-risk mandatory zones, notably dynamic speed reduction areas in the Gulf of St. Lawrence, require enhanced monitoring and extended buffer zones. Critical gaps persist in unregulated high-traffic regions intersecting whale habitats: North Atlantic shipping corridors, Northeast Asian waters (Japan/Korean Peninsula), and the Cape of Good Hope maritime zone. These oversight areas, characterized by dense port networks and cetacean activity, urgently demand standardized regulatory frameworks integrating adaptive speed controls, mandatory observation protocols, and real-time whale detection systems to mitigate escalating anthropogenic pressures.

Emerging research demonstrates the strategic value of IMMAs in mitigating ship strike risks, as exemplified by Santa Barbara Channel, California (USA), case studies [61]. These initiatives highlight the critical role of habitat-centric conservation in safeguarding endangered species and maintaining biodiversity. This systematic analysis of maritime pressures across MPAs, EBSAs, and IMMAs establishes a scientific foundation for targeted species protection, enabling policymakers to prioritize regulatory interventions in high-risk zones where shipping corridors intersect critical marine habitats.

## 5. Conclusions

This study systematically evaluated shipping pressures from global maritime activities on critical marine habitats, revealing concentrated shipping impacts in coastal zones. Shipping pressure was unevenly distributed across global waters, with coastal waters (EEZs specifically) showing higher shipping pressure and greater vulnerability. Whale collision risk assessment corroborated these patterns, identifying multi-species hotspots (>6 species) in the North Atlantic, Gulf of St. Lawrence, and Northeast Asian marginal seas, with high-risk zones lacking regulatory frameworks. These findings underscore the urgent need for adaptive, multi-species management strategies. Mandatory speed restrictions and dynamic routing in high-overlap zones could be carried out, whereas unregulated regions (e.g., Cape of Good Hope, Arctic emerging routes) require standardized measures integrating real-time monitoring and expanded buffer zones. Climate-driven cetacean migrations and expanding Arctic shipping point to the necessity for proactive habitat protection in polar refuges. Incorporating future shifts in whale distribution and shipping routes would provide more forward-looking insights for conservation. Critically, this study highlights the limitations of area-averaged pressure assessments in vast EBSAs, advocating for micro-scale hotspot identification to safeguard discrete critical habitats. In addition, since species have unique reactions and behavioral habits in response to ship traffic, collision risk assessments that take into account species behavior patterns and differences in vulnerability will further improve the accuracy of the assessments. By aligning maritime spatial planning with IUCN species recovery goals, this work provides a replicable framework to reconcile global trade demands with the preservation of marine biodiversity across jurisdictional boundaries.

## Figures and Tables

**Table 1:** Units, resolution, and sources of 11 environmental factors.

Variable	Units	Spatial Resolution	Source
Sea Surface Temperature (SST)	°C	0.25°	Cpernicus ^1^
Salinity (Sal)	-	0.25°	Cpernicus ^1^
Chlorophyll (Chl)	mg/m^3^	0.25°	Cpernicus ^1^
Mixed Layer Depth (MLD)	m	0.25°	Cpernicus ^1^
Sea Ice Thickness (SIT)	m	0.25°	Cpernicus ^1^
Sea Ice Cover (SIC)	m	0.25°	Cpernicus ^1^
Sea Surface Height (SSH)	Fraction	0.25°	Cpernicus ^1^
Bathymetry (Bat)	m	0.05°	Bio-ORACLE ^2^
Topographic Slope (TS)	-	0.05°	Bio-ORACLE ^2^
Topographic Aspect (TA)	-	0.05°	Bio-ORACLE ^2^
Land from Distance (LandD)	km	0.08°	MARSPEC ^3^

^1^ COPERNICUS (https://www.copernicus.eu/); ^2^ Bio-ORACLE v3.0 (https://bio-oracle.org/) [29]; ^3^ MARSPEC (https://doi.org/10.6084/m9.figshare.c.3305499.v1 accessed on 26 February 2024) [30].

## Data Availability

The dataset supporting the findings of this research is available upon request.

## Supplementary Materials

The following supporting information can be downloaded at https://www.mdpi.com/article/10.3390/ani15142144/s1, Text S1: Detailed description of the grading of shipping pressures; Table S1: Levels of shipping pressure and the ocean area covered by each shipping pressure level; Table S2: Values of the evaluation metrics for the ensemble model for each species; Table S3: The spatial overlap ratio of whale collision hotspots; Figure S1: Distribution of Marine Protected Areas (A), Ecologically or Biologically Significant Marine Areas (B), and Important Marine Mammals Areas (C); Figure S2: Pearson correlation analysis of environmental factors; Figure S3: Relative importance of 9 environmental factors for model construction; Figure S4: Continuous distributions predicted by SSDM for nine species of whales; Figure S5: Distribution of hotspots (≥90%, ≥95%, ≥99%, ≥99.5%) of whale–vessel collisions.

## References

[1] United Nations Conference on Trade and Development (2021). Review of Maritime Transport. https://unctad.org/system/files/official-document/rmt2021_en_0.pdf.

[2] Tournadre J. (2014). Anthropogenic pressure on the open ocean: The growth of ship traffic revealed by altimeter data analysis. Geophys. Res. Lett..

[3] Pirotta V., Grech A., Jonsen I.D., Laurance W.F., Harcourt R.G. (2018). Consequences of global shipping traffic for marine giants. Front. Ecol. Environ..

[4] Bereza D., Shenkar N. (2022). Shipping voyage simulation reveals abiotic barriers to marine bioinvasions. Sci. Total Environ..

[5] Carter E.E., Tregenza T., Stevens M. (2020). Ship noise inhibits colour change, camouflage, and anti-predator behaviour in shore crabs. Curr. Biol..

[6] Escajeda E.D., Stafford K.M., Woodgate R.A., Laidre K.L. (2023). Quantifying the effect of ship noise on the acoustic environment of the bering strait. Mar. Pollut. Bull..

[7] Schoeman R.P., Patterson-Abrolat C., Plön S. (2020). A global review of vessel collisions with marine animals. Front. Mar. Sci..

[8] Quaglia I., Visioni D. (2024). Modeling 2020 regulatory changes in international shipping emissions helps explain anomalous 2023 warming. Earth Syst. Dyn..

[9] Arzaghi E., Sajid Z., Abbassi R. (2020). Chapter 10—Advanced methods for environmental risk assessment in offshore operations. Methods Chem. Process Saf..

[10] Hulme P.E. (2021). Unwelcome exchange: International trade as a direct and indirect driver of biological invasions worldwide. One Earth.

[11] Frankish C.K., von Benda-Beckmann A.M., Teilmann J., Tougaard J., Dietz R., Sveegaard S., Binnerts B., de Jong C.A.F., Nabe-Nielsen J. (2023). Ship noise causes tagged harbour porpoises to change direction or dive deeper. Mar. Pollut. Bull..

[12] Grorud-Colvert K., Sullivan-Stack J., Roberts C., Constant V., Costa B.H.E., Pike E.P., Kingston N., Laffoley D., Sala E., Claudet J. (2021). The MPA Guide: A framework to achieve global goals for the ocean. Science.

[13] Clark M.R., Rowden A.A., Schlacher T.A., Guinotte J., Dunstan P.K., Williams A., O’Hara T.D., Watling L., Niklitschek E., Tsuchida S. (2014). Identifying Ecologically or Biologically Significant Areas (EBSA): A systematic method and its application to seamounts in the South Pacific Ocean. Ocean Coast. Manag..

[14] Corrigan C.M., Ardron J.A., Comeros-Raynal M.T., Hoyt E., Di Sciara G.N., Carpenter K.E. (2014). Developing important marine mammal area criteria: Learning from ecologically or biologically significant areas and key biodiversity areas. Aquat. Conserv..

[15] UNEP-WCMC, IUCN Protected Planet: The World Database on Protected Areas (WDPA) and World Database on Other Effective Area-based Conservation Measures (WD-OECM). https://www.protectedplanet.net.

[16] Roman J., Estes J.A., Morissette L., Smith C., Costa D., McCarthy J., Nation J., Nicol S., Pershing A., Smetacek V. (2014). Whales as marine ecosystem engineers. Front. Ecol. Environ..

[17] Cates K., Demaster D.P., Brownell R., Silber G. Strategic Plan to Mitigate the Impacts of Ship Strikes on Cetacean Populations: 2017–2020. https://www.researchgate.net/publication/332539367_Strategic_Plan_to_Mitigate_the_Impacts_of_Ship_Strikes_on_Cetacean_Populations_2017-2020.

[18] Nisi A.C., Welch H., Brodie S., Leiphardt C., Rhodes R., Hazen E.L., Redfern J.V., Branch T.A., Barreto A.S., Calambokidis J. (2024). Ship collision risk threatens whales across the world’s oceans. Science.

[19] Kraus S.D., Brown M.W., Caswell H., Clark C.W., Fujiwara M., Hamilton P.K., Kenney R.D., Knowlton A.R., Landry S., Mayo C.A. (2005). North atlantic right whales in crisis. Science.

[20] Thomas P.O., Reeves R.R., Brownell R.L. (2015). Status of the world’s baleen whales. Mar. Mammal Sci..

[21] Rockwood R.C., Calambokidis J., Jahncke J. (2017). High mortality of blue, humpback and fin whales from modeling of vessel collisions on the U.S. West coast suggests population impacts and insufficient protection. PLoS ONE.

[22] Crum N.J., Gowan T.A., Krzystan A., Martin J. (2019). Quantifying risk of whale–vessel collisions across space, time, and management policies. Ecosphere.

[23] Flanders Marine Institute Global Oceans and Seas, Version 1. https://www.marineregions.org/.

[24] Aiello-Lammens M.E., Boria R.A., Radosavljevic A., Vilela B., Anderson R.P. (2015). Spthin: An R package for spatial thinning of species occurrence records for use in ecological niche models. Ecography.

[25] Breen P., Brown S., Reid D., Rogan E. (2016). Modelling cetacean distribution and mapping overlap with fisheries in the northeast Atlantic. Ocean Coast. Manag..

[26] Correia A.M., Sousa-Guedes D., Gil Á., Valente R., Rosso M., Sousa-Pinto I., Sillero N., Pierce G.J. (2021). Predicting cetacean distributions in the eastern north Atlantic to support marine management. Front. Mar. Sci..

[27] Sun B., Zhao L., Shao F., Lu Z., Tian J., Liu C. (2022). Estimating the impacts of climate change on the habitat suitability of common minke whales integrating local adaptation. Front. Mar. Sci..

[28] Dormann C.F., Elith J., Bacher S., Buchmann C., Carl G., Carré G., Marquéz J.R.G., Gruber B., Lafourcade B., Leitão P.J. (2013). Collinearity: A review of methods to deal with it and a simulation study evaluating their performance. Ecography.

[29] Assis J., Bejarano S.J.F., Salazar V.W., Schepers L., Gouvêa L., Fragkopoulou E., Leclercq F., Vanhoorne B., Tyberghein L., Serrão E.A. (2024). Bio-ORACLE v3.0. Pushing marine data layers to the CMIP6 Earth System Models of climate change research. Glob. Ecol. Biogeogr..

[30] Sbrocco E.J., Barber P.H. (2013). MARSPEC: Ocean climate layers for marine spatial ecology. Ecology.

[31] Schmitt S., Pouteau R., Justeau-Allaire D., de Boissieu F., Birnbaum P. (2017). SSDM: An R package to predict distribution of species richness and composition based on stacked species distribution models. Methods Ecol. Evol..

[32] Thuiller W., Lafourcade B., Engler R., Araújo M.B. (2009). BIOMOD—A platform for ensemble forecasting of species distributions. Ecography.

[33] Naimi B., Araújo M.B. (2016). sdm: A reproducible and extensible R platform for species distribution modelling. Ecography.

[34] Barbet-Massin M., Jiguet F., Albert C.H., Thuiller W. (2012). Selecting pseudo-absences for species distribution models: How, where and how many?. Methods Ecol. Evol..

[35] Guisan A., Thuiller W., Zimmermann N.E. (2017). Habitat Suitability and Distribution Models: With Applications in R.

[36] Castellanos A.A., Huntley J.W., Voelker G., Lawing A.M. (2019). Environmental filtering improves ecological niche models across multiple scales. Methods Ecol. Evol..

[37] Marmion M., Saarimaa M., Luoto M., Heikkinen R.K., Thuiller W. (2009). Evaluation of consensus methods in predictive species distribution modeling. Divers. Distrib..

[38] Yang W., Sun S., Wang N., Fan P., You C., Wang R., Zheng P., Wang H. (2023). Dynamics of the distribution of invasive alien plants (*Asteraceae*) in China under climate change. Sci. Total Environ..

[39] UNCTAD Key Statistics and Trends in International Trade 2024. https://unctad.org/publication/key-statistics-and-trends-international-trade-2024.

[40] Stanley J.A., Van Parijs S.M., Davis G.E., Sullivan M., Hatch L.T. (2021). Monitoring spatial and temporal soundscape features within ecologically significant U.S. National Marine Sanctuaries. Ecol. Appl..

[41] Verschuur J., Koks E.E., Hall J.W. (2022). Ports’ criticality in international trade and global supply-chains. Nat. Commun..

[42] Qi X., Li Z., Zhao C., Zhang Q., Zhou Y. (2024). Environmental impacts of Arctic shipping activities: A review. Ocean Coast. Manag..

[43] Yang L.K., Jiang M.M. (2024). The impact of opening the Arctic Northeast Passage on China’s carbon emissions. Transp. Policy.

[44] Shang L., Hu Z., Deng Y., Li J., Liu Y., Song X., Zhai X., Zhan Z., Tian W., Xu J. (2024). Transoceanic ships as a source of alien dinoflagellate invasions of inland freshwater ecosystems. Harmful Algae.

[45] Miljutin D.M., Miljutina M.A., Arbizu P.M., Galéron J. (2011). Deep-sea nematode assemblage has not recovered 26 years after experimental mining of polymetallic nodules (Clarion-Clipperton Fracture Zone, Tropical Eastern Pacific). Deep Sea Res. Part I Oceanogr. Res. Pap..

[46] Galli M., Tepsich P., Baini M., Panti M.R., Vafeiadou A., Pantelidou M., Moulins A., Fossi M.C. (2022). Microplastic abundance and biodiversity richness overlap: Identification of sensitive areas in the Western Ionian Sea. Mar. Pollut. Bull..

[47] Başkale E., Sözbilen D., Katılmış Y., Azmaz M., Kaska Y. (2018). An evaluation of sea turtle strandings in the Fethiye-Göcek Specially Protected Area: An important foraging ground with an increasing mortality rate. Ocean Coast. Manag..

[48] Lin H.J., Hsu C.B., Liao S.H., Chen C.P., Hsieh H.L. (2015). Effects of *Spartina alterniflora* invasion on the abundance and community of Meiofauna in a subtropical wetland. Wetlands.

[49] Xue X., Xue J., Liu W., Adams D.H., Kannan K. (2017). Trophic magnification of parabens and their metabolites in a subtropical marine food web. Environ. Sci. Technol..

[50] Barwick M., Maher W. (2003). Biotransference and biomagnification of selenium copper, cadmium, zinc, arsenic and lead in a temperate seagrass ecosystem from lake Macquarie Estuary, NSW, Australia. Mar. Environ. Res..

[51] Li H., Zhang Z., Sun Y., Wang W., Xie J., Xie C., Hu Y., Gao Y., Xu X., Luo X. (2021). Tetrabromobisphenol A and hexabromocyclododecanes in sediments and biota from two typical mangrove wetlands of South China: Distribution, bioaccumulation and biomagnification. Sci. Total Environ..

[52] Owen K., Carlström J., Eriksson P., Andersson M., Nordström R., Lalander E., Sveegaard S., Kygn L.A., Griffiths E.T., Cosentino M. (2024). Rerouting of a major shipping lane through important harbour porpoise habitat caused no detectable change in annual occurrence or foraging patterns. Mar. Pollut. Bull..

[53] Wang Z.T., Supin A.Y., Akamatsu T., Duan P., Yang Y., Wang K., Wang D. (2021). Auditory evoked potential in stranded melon-headed whales (*Peponocephala electra*): With severe hearing loss and possibly caused by anthropogenic noise pollution. Ecotoxicol. Environ. Saf..

[54] Farmer N.A., Baker K., Zeddies D.G., Denes S.L., Noren D.P., Garrison L.P., Machernis A., Fougères E.M., Zykov M. (2018). Population consequences of disturbance by offshore oil and gas activity for endangered sperm whales (*Physeter macrocephalus*). Biol. Conserv..

[55] Guzman H., Gomez C., Guevara C.A., Kleivane L. (2012). Potential vessel collisions with Southern Hemisphere humpback whales wintering off Pacific Panama. Mar. Mammal Sci..

[56] Van Der Hoop J.M., Vanderlaan A.S.M., Taggart C.T. (2012). Absolute probability estimates of lethal vessel strikes to North Atlantic right whales in Roseway Basin, Scotian Shelf. Ecol. Appl..

[57] Vanderlaan A.S.M., Taggart C.T. (2009). Efficacy of a voluntary area to be avoided to reduce risk of lethal vessel strikes to endangered whales. Conserv. Biol..

[58] International Whaling Commission Strategic Plan to Mitigate the Impacts of Ship Strikes on Cetacean Populations: 2022–2032. https://archive.iwc.int/pages/download.php?direct=1&noattach=true&ref=19858&ext=pdf.

[59] World Wildlife Fund Arctic Blue Corridors Report (2024). Safeguarding Migrating Whales from Growing Pressures for a Connected Arctic Ocean..

[60] World Shipping Council WSC Whale Chart: A Global Voyage Planning Aid to Protect Whales. https://safety4sea.com/wp-content/uploads/2024/10/WSC-Whale-Chart-Oct_24.pdf.

[61] Agardy T., Cody M., Hastings S., Hoyt E., Nelson A., Tetley M., Sciara G.N.D. (2019). Looking beyond the horizon: An early warning system to keep marine mammal information relevant for conservation. Aquat. Conserv..

