# Supplementary material for: Global Hotspots of Whale–Ship Collision Risk: A Multi-Species Framework Integrating Critical Habitat Zonation and Shipping Pressure for Conservation Prioritization
Source: Animals (Basel). 2025 Jul 20;15(14):2144. doi: 10.3390/ani15142144 (PMC12291979; doi:10.3390/ani15142144)

Supplementary Information for Global assessment of maritime shipping pressures on critical marine habitats

Text S1 Detailed description of the grading of shipping pressures.

Maritime navigation poses multifaceted threats to marine ecosystems through greenhouse gas emissions, chemical and noise pollution, and physical collisions with megafauna [1]. Conventional risk assessments, which prioritize vessel-specific metrics (e.g., size, speed, heading), inadequately address cumulative risks in mixed-traffic environments. Existing research predominantly isolates individual impacts such as ship strikes or emissions, leaving a critical gap in holistic risk evaluation. Under the BBNJ framework, this study establishes a methodology to quantify ecosystem pressures across shipping density gradients, integrating collision risks and voyage duration data from Global Fishing Watch.

We concluded that the more active the shipping activity, the more stressful it becomes as ship voyage duration increases. Given the absence of established thresholds for irreversible ecosystem damage, we adopted a precautionary approach by defining high-stress zones as regions within the top 1% of global shipping duration (99th percentile) [2]. Due to the discontinuous hierarchical structure of voyage duration data (Level 1-7 in Figure 1), we identified Classes 5-8 (0.944% of ocean area) as high-stress zones, effectively capturing dense shipping corridors while maintaining ecological relevance. Expanding this threshold to Class 4 would encompass 7.275% of global waters, exceeding the indicative 1% benchmark. Low-stress areas (Level 1-3) were defined by annual voyage durations <100 hours (<1 hour/day), with remaining classes classified as medium stress. This tiered classification balances scientific rigor with practical applicability for marine spatial planning.

Reference:

[1] Schoeman, R.P.; Patterson-Abrolat, C.; Plön, S. A global review of vessel collisions with marine animals. *Front. Mar. Sci.* **2020**, *7*. https://doi.org/10.3389/fmars.2020.00292

[2] Nisi, A.C.; Welch, H.; Brodie, S.; Leiphardt, C.; Rhodes, R.; Hazen, E.L.; Redfern, J.V.; Branch, T.A.; Barreto, A.S.; Ca-lambokidis, J.; Clavelle, T.; Dares, L.; de Vos, A.; Gero, S.; Jackson, J.A.; Kenney, R.D.; Kroodsma, D.; Leaper, R.; Mccauley, D.J.; Moore, S.E.; Ovsyanikova, E.; Panigada, S.; Robinson, C.V.; White, T.; Wilson, J.; Abrahms, B. Ship collision risk threatens whales across the world's oceans. *Science* **2024**, *386*, 870-875. https://doi.org/10.1126/science.adp1950

Table S1. Levels of shipping pressure and the ocean area covered by each shipping pressure level.

Shipping pressure level	Shipping hour (h/grid)	Area (%)
1	0–1	15.579
2	1–10	42.798
3	$10–10^2$	34.347
4	$10^2–10^3$	6.331
5	$10^3–10^4$	0.819
6	$10^4–10^5$	0.117
7	$10^5–10^6$	0.008

Table S2. Values of evaluation metrics for the ensemble model for each species. AUC: area under the curve of receiver operating characteristic; TSS: true skill statistic.

ESDM	AUC	TSS
Balaena mysticetus	0.963	0.857
Balaenoptera acutorostrata	0.928	0.727
Balaenoptera borealis	0.925	0.723
Balaenoptera musculus	0.939	0.744
Balaenoptera physalus	0.922	0.727
Eschrichtius robustus	0.974	0.893
Eubalaena glacialis Borowski	0.974	0.902
Megaptera novaeangliae	0.933	0.744
Physeter macrocephalus	0.933	0.720

Table S3. The spatial overlap ratio of whale collision hotspots. It was quantified by dividing the overlapping area by the total hotspot area.

Num of species	Overlap ratio
1	43.5%
2	17.6%
3	15.7%
4	9.0%
5	7.5%
6	5.6%
7	1.1%

Figure S1. Distribution of Marine Protected Areas (A), Ecologically or Biologically Significant Marine Areas (B) and Important Marine Mammals Areas (C).

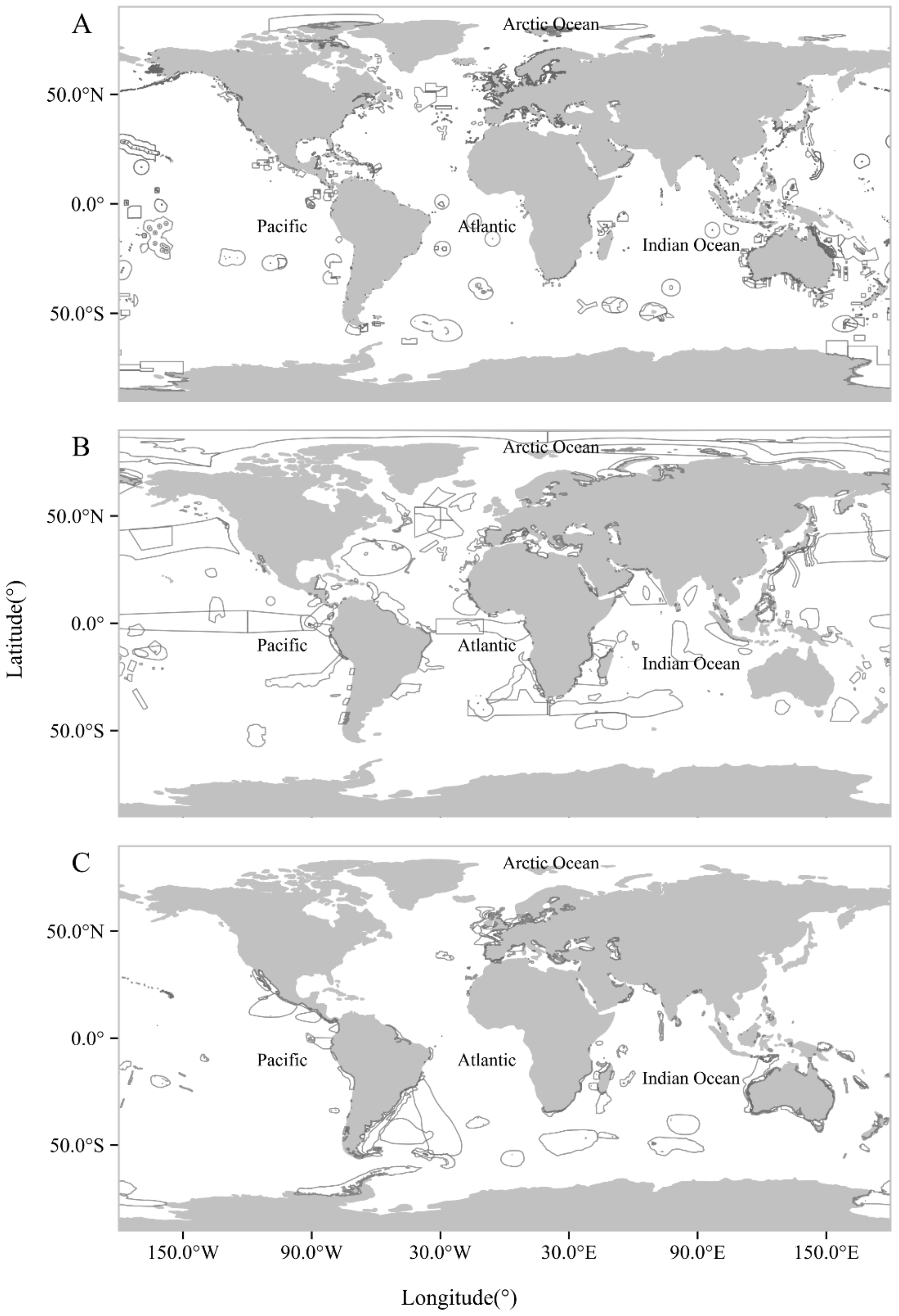

Figure S2. Pearson correlation analysis of environmental factors. Aspect: Topographic Aspect; Bat: Bathymetric; Chl: Chlorophyll; LandD: Distance from Shore; MLD: Mixed Layer Depth; SSH: Sea Surface Height; SIC: Sea Ice Concentration; SIT: Sea Ice Thickness; Slope: Topographic Slope; Sal: Salinity; SST: Sea Surface Temperature.

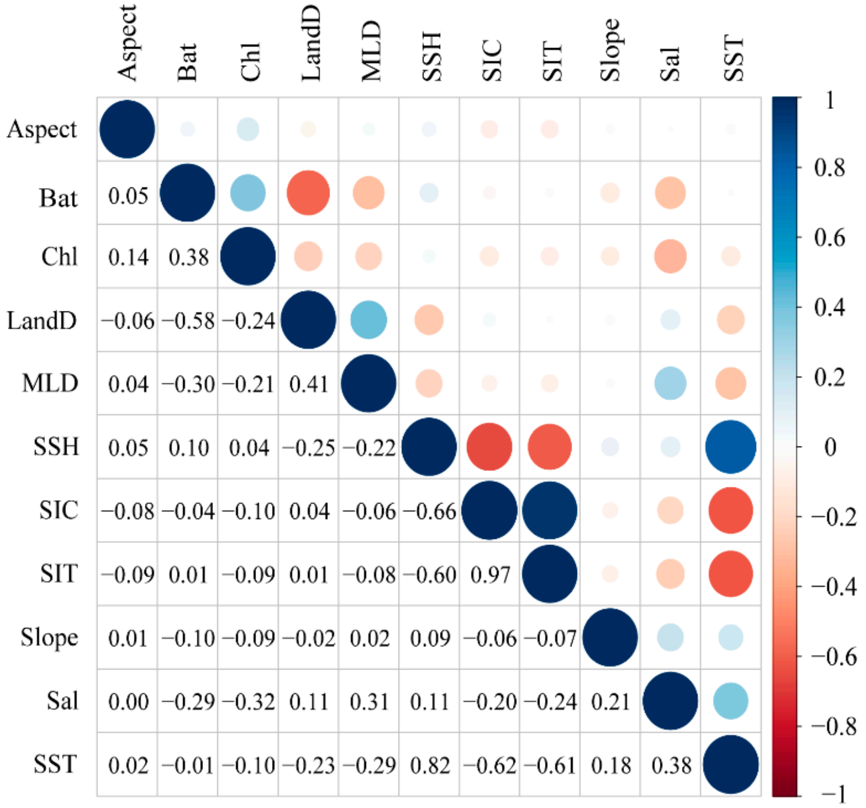

Figure S3. relative importance of 9 environmental factors for model construction. Aspect: Topographic Aspect; Bat: Bathymetric; Chl: Chlorophyll; LandD: Distance from Shore; MLD: Mixed Layer Depth; SIC: Sea Ice Concentration; Slope: Topographic Slope; Sal: Salinity; SST: Sea Surface Tempreature.

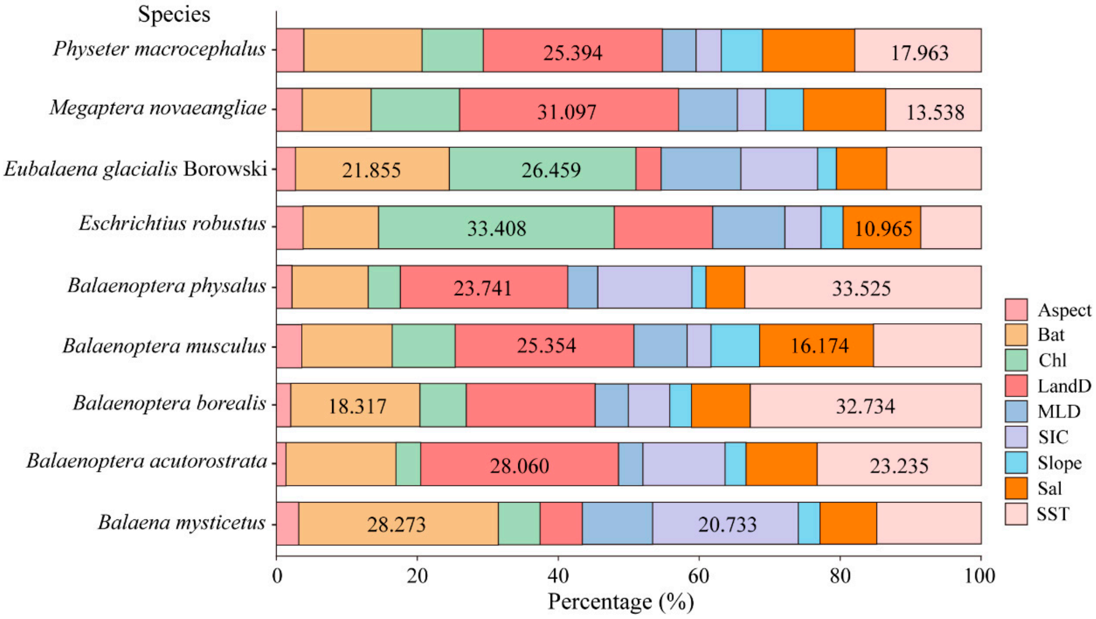

Figure S4. Continuous distributions predicted by the SSDM model for nine species of whales.

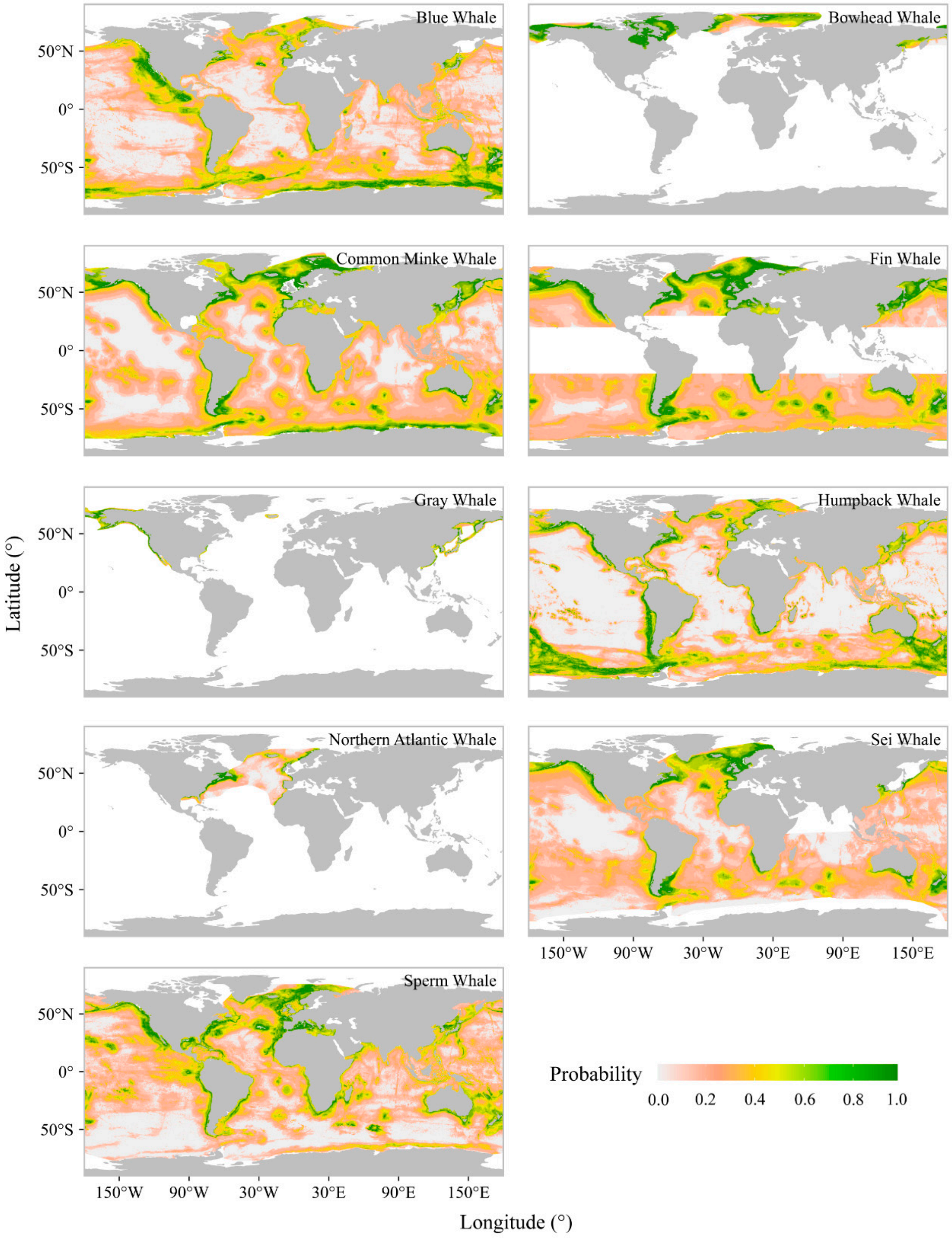

Figure S5. Distribution of hotspots (⩾90%, ⩾95%, ⩾99%, ⩾99.5%) of whale-vessel collisions.

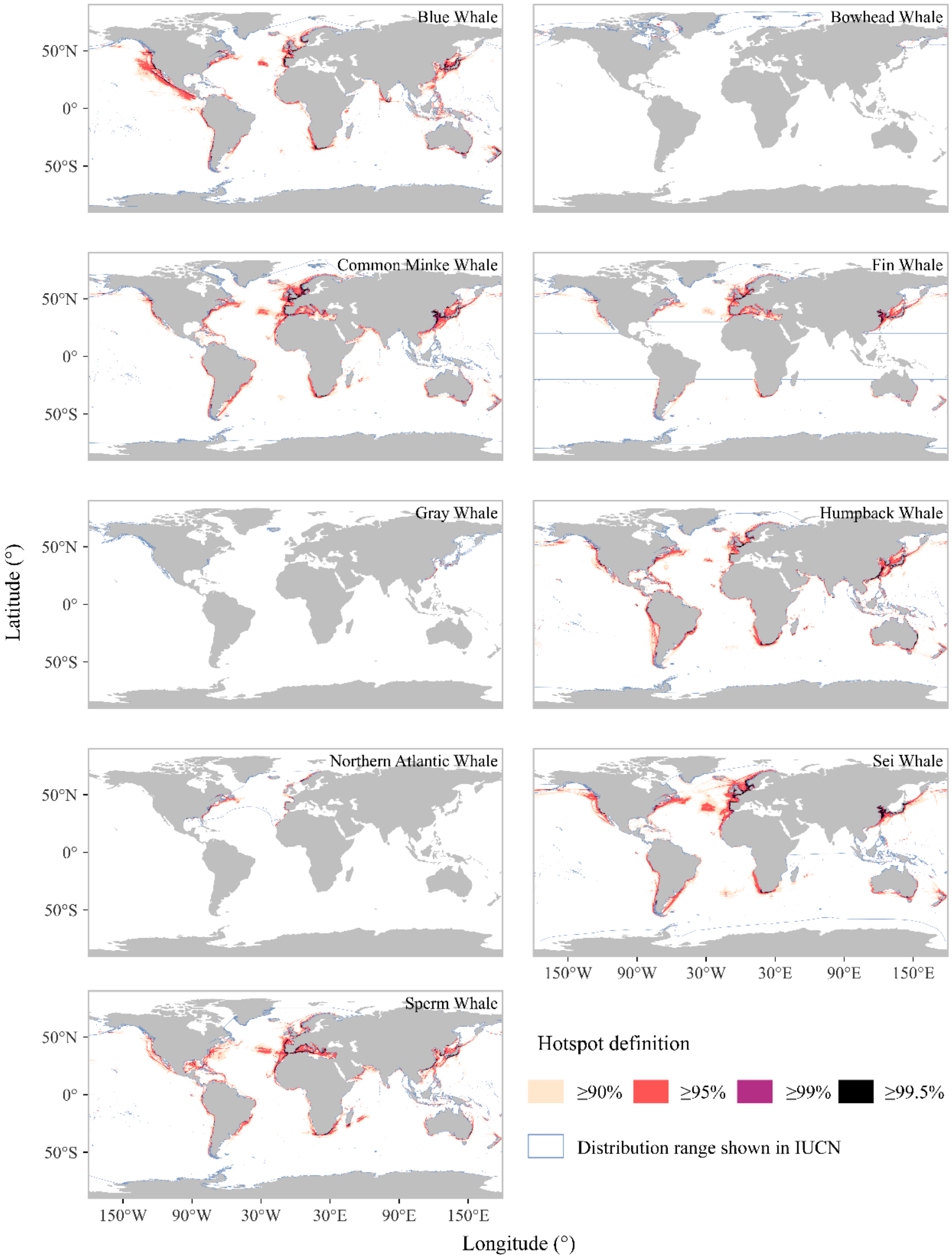

Supplement: Supplementary file 1 [file animals-15-02144-s001.zip › Supplementary Materials.pdf]
